# Cancer Exosomes as Conveyors of Stress-Induced Molecules: New Players in the Modulation of NK Cell Response

**DOI:** 10.3390/ijms20030611

**Published:** 2019-01-31

**Authors:** Elisabetta Vulpis, Alessandra Soriani, Cristina Cerboni, Angela Santoni, Alessandra Zingoni

**Affiliations:** 1 Department of Molecular Medicine, Sapienza University of Rome, Laboratory affiliated to Istituto Pasteur Italia—Fondazione Cenci Bolognetti, 00161 Rome, Italy; elisabetta.vulpis@uniroma1.it (E.V.); alessandra.soriani@uniroma1.it (A.S.); cristina.cerboni@uniroma1.it (C.C.); angela.santoni@uniroma1.it (A.S.); 2 IRCCS, Neuromed, 86077 Pozzilli, Italy

**Keywords:** NK cells, exosomes, NKG2D, DAMPs, immune surveillance, stress, cancer

## Abstract

Natural killer (NK) cells are innate lymphoid cells that play a pivotal role in tumor surveillance. Exosomes are nanovesicles released into the extracellular environment via the endosomal vesicle pathway and represent an important mode of intercellular communication. The ability of anticancer chemotherapy to enhance the immunogenic potential of malignant cells mainly relies on the establishment of the immunogenic cell death (ICD) and the release of damage-associated molecular patterns (DAMPs). Moreover, the activation of the DNA damage response (DDR) and the induction of senescence represent two crucial modalities aimed at promoting the clearance of drug-treated tumor cells by NK cells. Emerging evidence has shown that stress stimuli provoke an increased release of exosome secretion. Remarkably, tumor-derived exosomes (Tex) produced in response to stress carry distinct type of DAMPs that activate innate immune cell populations. Moreover, stress-induced ligands for the activating receptor NKG2D are transported by this class of nanovesicles. Here, we will discuss how Tex interact with NK cells and provide insight into their potential role in response to chemotherapy-induced stress stimuli. The capability of some “danger signals” carried by exosomes that indirectly affect the NK cell activity in the tumor microenvironment will be also addressed.

## 1. Introduction

Cellular cross-talk is a crucial event in multicellular organisms, where cells can communicate with each other through direct cell–cell contact or through the release of soluble factors. Exosomes are nanovesicles released into the extracellular environment via the endosomal vesicle pathway by fusion with the plasma membrane and are essential for intercellular communication [1]. In the tumor microenvironment, the content of cancer-secreted exosomes can be transferred not only to the neoplastic cells but also to different type of immune cells, thus modulating the anti-tumor immune response and affecting tumor progression [2]. 

Natural killer (NK) cells are innate lymphoid cells [3] that play a pivotal role in tumor surveillance through both the direct killing of cancer cells and cytokine production [4]. NK cell activation is tightly regulated by a delicate balance between activating and inhibitory signals, with the latter being primarily transduced by receptors for Major Histocompatibility Complex (MHC) class I molecules (KIRs, CD94/NKG2A). Recognition of induced self on tumor cells triggers a number of non-MHC class I–restricted activating receptors, such as NK group 2D (NKG2D), DNAX accessory molecule-1 (DNAM-1/CD226), and the natural cytotoxicity receptors (NCRs) [5]. Moreover, NK cells can mediate target cell death through the surface expression of death inducing ligands belonging to the tumor necrosis factor (TNF) family, such as Fas ligand (FasL) and TNF-related apoptosis inducing ligand (TRAIL). 

The role of tumor-derived exosomes (Tex) on the modulation of NK cell-mediated functions is still a matter of debate and seems to be dependent on the molecular cargo and the source of these vesicles [6]. 

The failure of antitumor immunity is often due to low immunogenicity of cancer cell variants or to the aptitude of neoplastic cells to induce immunosuppression. The fulfillment of anticancer therapies to enhance the immunogenic potential of malignant cells is based on different mechanisms, including the activation of the DNA damage response (DDR) and the induction of senescence as two crucial modalities promoting the clearance of drug-treated tumor cells by NK cells. In this context, low doses of chemotherapeutic drugs have been shown to induce immunogenic senescence and stimulate NK cell-mediated recognition and clearance of drug-treated tumor cells via the upregulation of NKG2D and DNAM-1 activating ligands on the surface of cancer cells [7,8,9,10,11]. In addition, the establishment of the immunogenic cell death (ICD) and the release of damage-associated molecular patterns (DAMPs) represent another important approach to strengthen the efficacy of immunotherapy [12]. DAMPs are endogenous molecules harbored intracellularly in normal conditions, but they can be exposed on the tumor cell surface or released upon stress, injury, or cell death, thereby becoming able to bind to cognate receptors on immune cells [13,14,15]. Thus, DAMPs can directly activate innate immune cells, such as the Dendritic cells (DCs), macrophages, neutrophils and NK cells, and indirectly stimulate the adaptive T cell responses by promoting maturation of DCs and tumor antigen processing and presentation. 

Emerging evidence has shown the presence of different types of DAMPs in exosomes, including molecules belonging to the heat shock protein (HSP) family [16,17,18], and the high-mobility group box 1 (HMGB1) [19,20], but also dsDNA [21,22] and RNA [23], all of which are able to engage distinct pattern recognition receptors (PRRs). Of interest, stress-induced ligands for the NKG2D activating receptor have also been reported to be associated with exosomes [24,25].

Herein, we will discuss how cancer-derived exosomes contribute to regulate the NK cell-mediated functions in response to chemotherapeutic treatment, as well as in the presence of stress stimuli focusing on: (i) the modulation of exosome release from cancer cells under stress conditions; and (ii) the stress-induced molecules associated with cancer-derived exosomes, such as DAMPs and NKG2D ligands.

## 2. Exosomes, General Features

Extracellular vesicles (EVs) are a heterogeneous group of bilayer membrane particles that can be classified into three subtypes according to the cellular compartment they originate from and their size. Specifically, apoptotic bodies having a size falling in the range of 1000–5000 nm represent the largest class of vesicles and are generated during apoptotic cell death; microvesicles or ectosomes (200–1000 nm) originate through the budding of the plasma membrane; and, finally, the exosomes representing the smallest type of EVs are characterized by a size ranging between 30–150 nm and are formed in the late endosomal compartment [26]. 

The present review focuses mainly on exosomes that contain proteins, nucleic acids (DNA, mRNA and miRNAs), lipids and metabolites. These nanovesicles, initially considered only a way to expel cellular garbage, have generate significant interest in recent decades because of their ability to carry and “protect” biologically active molecules in the extracellular environment and to transfer them to target cells. Exosomes are released from almost all cell types under both physiological and pathological conditions, such as cancer. In this condition, tumor exosomes (Tex) can interact and be taken up by cancer cells themselves or by other cells present in the tumor microenvironment or distant from the tumor site, causing different effects. In fact, they are able to modulate tumor-induced immune response, angiogenesis, tumor progression, and premetastatic niche formation in an autocrine, paracrine or endocrine manner thanks to their ability to move throughout the body fluids [27,28]. 

From a biochemical point of view, exosomes are structurally composed by a lipid bilayer membrane enriched in cholesterol, phosphatidylserine, sphingomyelin, ceramide, sphingolipids and a low amount of phosphatidylcholine [29]. Moreover, these nanovesicles typically express enriched sets of proteins that comprise some members of the tetraspanin family (i.e., CD9, CD63, CD81), adhesion molecules, cytoskeleton components, endosomal sorting complexes required for transport (ESCRT complex) (TSG101, ALIX), HSPs, annexins and Rab proteins. Interestingly, several studies have shown that the majority of exosomal proteins are loaded through various sorting mechanisms. For example, post-translational modifications, including monoubiquitylation, glycosylation, sumoylation, oxidation and phosphorylation, regulate the exosomal cargo targeting the proteins into the multivesicular bodies [30]. The enrichment of specific proteins into exosomes is also strongly dependent on the origin of the parental cell. Emerging evidence has shown that exosome composition, as well as biogenesis and secretion, can be affected by external stimuli, including stress conditions such as heat shock, oxidative stress, chemotherapy, irradiation, hypoxia, and hypothermia.

## 3. Modulation of Tex Release in Response to Stress Stimuli

A number of studies have shown that different stress conditions provoke increased exosome release from cancer cells. Interestingly, thermal and oxidative stress enhance the exosome secretion from leukemia/lymphoma T and B cell lines [31] and hypoxic conditions have been shown to be effective to enhance Tex release from breast cancer cells [32]. In addition, sublethal doses of various chemotherapeutic agents, including genotoxic drugs and proteasome inhibitors, stimulate exosome secretion in different tumor models. As such, multiple myeloma cells released an augmented number of nanovescicles upon melphalan [33,34] or bortezomib treatment [34]. Similarly, 5-fluorouracil, cisplatin and doxorubicin induced an increase in the amount of HSP70^+^ exosomes from melanoma and colon cancer cell lines [35]. Interestingly, these authors further proved that cisplatin treatment of mice-bearing tumors determined a huge increase of exosomes in the blood of drug-treated mice [35]. A recent study indicated that heat stress increased the quantity of doxorubicin-containing exosomes from tumor cells, and enhanced the anti-tumor effect of exosomes from the doxorubicin-treated tumor cells, suggesting new strategies for cancer therapy by the combined use of chemotherapy and hyperthermia [36]. 

Mechanisms behind the stress-induced exosome secretion are still largely unknown, although the contribution of the tumor-suppressor gene p53 has been described in different models. In this regard, Lehmann and coworkers have shown that irradiation of prostate cancer cells induced an augmented secretion of exosome-like vesicles with a mechanism mediated by p53 activation [37]. In addition, a p53-regulated gene product, the tumor suppressor activated pathway 6 (TSAP6), was shown to enhance exosome production in cells undergoing a p53 response to stress [38]. In line with these observations, exosome secretion was reported to be severely compromised in TSAP6-null mice [39]. Several studies have shown that increased exosome secretion in cancer cells can be associated to a senescent phenotype [37,40]. 

## 4. Tex as Carriers of Stress-Induced Molecules and DAMPs

As already mentioned, exosomes are released from cancer cells in the tumor microenvironment and their content can be transferred not only to the neoplastic cells but also to cells of the immune system. The exosome uptake can be mediated by various mechanisms such as protein–protein interaction, passive fusion with the plasma membrane through lipid–lipid interaction, and endocytosis, and they differ depending on the recipient cell [41,42]. Various molecules have been described to be involved in the interaction between exosomes and target cells, including integrins, immunoglobulins, proteoglycans and lectins, and these interactions appear to facilitate the endocytosis process and are important to define the selectivity of target cells [43]. Hwang and colleagues have shown that Intercellular adhesion molecule 1/Lymphocyte antigen-associated antigen-1 (ICAM-1/LFA-1) interactions are involved in the DC-derived exosome uptake by T lymphocytes [44]. A number of studies have described that Tex can be taken up by all leukocyte subpopulations, including NK cells, but the mechanisms behind this process are still largely unknown [45]. Our group has recently found that multiple myeloma-derived exosomes are taken up by human primary NK cells through a mechanism mainly dependent on endocytic routes requiring dynamin and caveolae/raft endocytosis [33]. The source of exosomes can strongly affect the efficiency of NK cell uptake as shown by the usage of exosomes derived from distinct cancer cell lines [46].

In the following sections, we will illustrate the role of some molecules acting as “danger signals”, as well as the stress-induced NKG2D ligands associated to Tex in the modulation of NK-cell mediated functions. The capability of some danger signals carried by exosomes that indirectly affect the NK cell activity in the tumor microenvironment will be also discussed.

### 4.1. NKG2D Ligands

The activating receptor NKG2D is a C-type lectin-like receptor expressed on NK cells, γδ T cells, CD8^+^ T cells, and a subset of CD4^+^ T cells, and represents a major recognition receptor for the detection and elimination of transformed cells. Engagement of NKG2D by its ligands on target cells triggers cytotoxicity and cytokine production. NKG2D ligands belong to the Retinoic Acid Early Inducible-1 gene, RAE (α−ε), H60 (a–c) and murine UL16-binding protein-like transcript, MULT1 families in mice and to the MHC-related genes, MIC (MICA and MICB) and UL16 binding proteins, ULBP (ULBP1–ULBP6) families in humans [47]. These molecules are generally absent in healthy cells, but are instead expressed on different type of cancer cells and can be induced or upregulated in response to an ample variety of stress stimuli [48]. In this regard, treatment of cancer cells with distinct classes of therapeutic drugs, such as genotoxic agents [7,49], histone deacetylase inhibitors [50,51], and proteasome inhibitors [52], upregulate NKG2D ligands on the surface of cancer cells favoring their NK cell recognition and killing [53]. NKG2D ligands can be released in the extracellular milieu through protease-mediated cleavage or associated with exosomes [54]. The choice of one of these processes is mainly dependent on the ligand type, as well as its allelic variant [55,56,57,58]. Recently, a number of studies have shown that NKG2D ligands from both MICA/B and ULBP families are expressed on the surface of exosome-like vesicles released from ovarian cancer [59], melanoma [60], and prostate cancer cells [61]. Remarkably, NKG2D ligands such as ULBP3 and ULBP1 [57], or the allelic variant MICA*008 [58], are secreted exclusively by exosomes.

As already mentioned, NKG2D ligand expression on the surface of cancer cells increases in response to stress stimuli, and as a consequence the amount of these molecules in exosomes could also be augmented. In line with these considerations, Hedlund and colleagues have shown that oxidative stress enhances the release of NKG2D ligand-bearing exosomes from cancer cell lines [31]. Differently from the metalloprotease-mediated shedding [49,62,63,64], it is still unclear whether the release of NKG2D ligands via exosomes also results in the reduction of their surface expression on cancer cells. 

**Figure 1:**
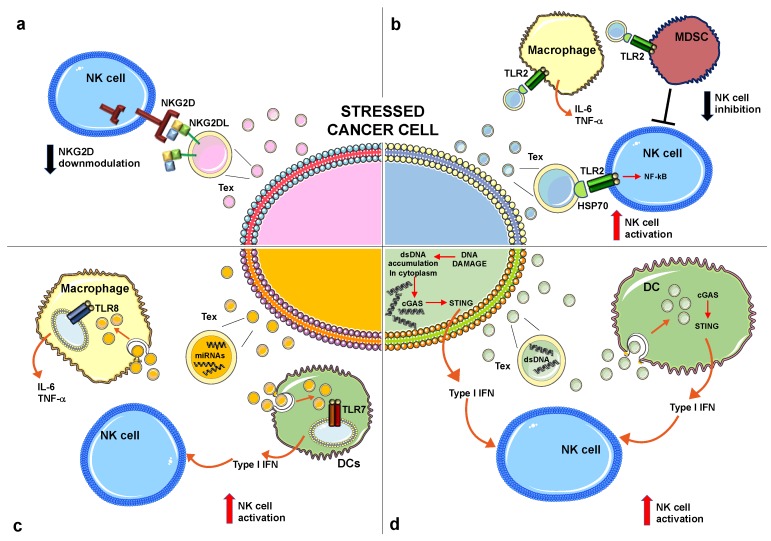
Effects of distinct molecules produced by stressed cancer cells on NK cell-mediated functions. (**a**) Exosomes expressing NKG2D ligands induce NKG2D downmodulation leading to an impairment of NKG2D-mediated cytotoxicity. (**b**) Exosomes expressing HSP70 engage TLR2 expressed on distinct innate immune cell populations as indicated. The direct TLR2 engagement on NK cells induces cellular activation. In contrast, TLR2 triggering on MDCSs induces the production of immunosuppressive factors that can impair NK cell activity. (**c**) Specific miRNAs can engage TLR8 expressed by macrophages leading to inflammatory cytokine production or TLR7 on plasmacytoid DCs leading to type I IFN production, that in turn contribute to NK cell activation. (**d**) Double strand DNA (dsDNA) accumulates in the cytoplasm of cancer cells in response to stress stimuli where it activates the cGAS/STING pathway leading to type I IFN production; in turn, dsDNA is packaged into exosomes and released in the tumor microenvironment where it is taken up by DCs that produce type I IFNs with a mechanism dependent on the cGAS/STING pathway. Type I IFNs, produced either by stressed cancer cells or DCs, activate NK cell-mediated functions, including cytotoxicity and cytokine production. “T” means inhibition. NK, Natural Killer; HSP70, Heat shock protein 70; NKG2D, NK group 2 member D; TLR2, Toll-like receptor 2; MDSCs, Myeloid suppressor cells; IFN, interferon; cGAS, Cyclic guanosine monophosphate (GMP)-adenosine monophosphate synthase; STING, stimulator of IFN gene.

It is likely that the expression of NKG2D ligands on the surface of tumor exosomes should preserve their biological activity by keeping the whole-molecule and the three-dimensional protein structure. It has been reported in different cellular models that exosomes expressing NKG2D ligands induced NKG2D downregulation in NK and CD8^+^ T cells leading to impaired cytotoxic function in vitro [58,59,61,65]. Of interest, although the NKG2D ligands associated with Tex down-regulate the cognate receptor, the constitutive levels of granzyme B and perforin in both cytotoxic T and NK cells are preserved [65]. On the other hand, Viaud and colleagues have shown that ULBP-1 expressed on the surface of DCs-derived exosomes directly engaged NKG2D and induced NK cell activation [66]. It is important to consider that NKG2D endocytosis not only leads to reduced cell-surface receptor levels but also controls signaling outcome in NK cells [67]. Thus, the possibility that exosome-associated NKG2D ligands could engage NKG2D, thereby triggering intracellular signaling, remains an intriguing and open question that requires further investigation (Figure 1a). 

### 4.2. The Heat Shock Protein Family

The heat shock proteins (HSPs) represent a class of chaperones that assist protein folding and prevent the formation of nonspecific protein aggregates, and are generally localized in intracellular compartments, such as cytoplasm, endoplasmic reticulum (ER) and mitochondria. HSPs are divided into five large families according to their molecular weight: HSP110, HSP90, HSP70, HSP60 and small HSPs [68]. A wide variety of stressful conditions can cause HSP mobilization to the plasma membrane or their release from cells, thus acting as potent danger signals. Several pieces of evidence demonstrate that extracellular-located HSPs can be associated with extracellular vesicles, including exosomes [69,70,71]. In general, exosomes expressing HSPs have immunostimulatory properties on NK cell-mediated functions. Notably, colon carcinoma derived HSP70 associated to exosomes stimulated NK cell migration and cytotoxic activity [72]. It has been demonstrated that the usage of anticancer drugs up-regulated the expression of distinct HSPs (e.g., HSP70, HSP60 and HSP90) on exosomes derived from human hepatocellular carcinoma cell lines. The authors further proved that HSP^+^ exosomes induced an increase of NK cell cytotoxic activity [73]. In addition, we have recently demonstrated that HSP70 on the surface of multiple myeloma-derived exosomes triggers NK cell-mediated IFN-γ production through a mechanism dependent on the activation of NF-κB signaling pathway by TLR2 engagement [33]. Of interest, HSP70^+^ exosomes were isolated from the bone marrow aspirates derived from multiple myeloma patients making evident that this class of nanovesicles is present in the tumor microenvironment, thus potentially contributing to the cross-talk between malignant plasma cells and immune cells [33]. The capability of HSP70 to bind to TLR2 determines the stimulation of other innate immune cell populations including macrophages, DCs, and myeloid-derived suppressor cells (MDSCs). Chow and colleagues have proposed a model of TLR2-mediated NF-κB activation and consequent inflammatory cytokine production in human macrophages in response to breast cancer cell-derived exosomes contributing to metastatic tumor development [74]. Other studies have reported that HSP70^+^ Tex are efficacious in the activation of MDSCs through TLR2 engagement leading to the production of immunosuppressive cytokines [35,75,76]. On the other hand, some evidence has shown the capacity of HSPs expressing exosomes to stimulate DCs leading to anti-tumor immune responses in distinct cancer models [77,78]. In this regard, multiple myeloma-derived exosomes overexpressing membrane-bound HSP70 have the capability to induce DC maturation and stimulate type 1 CD4^+^ T and CD8^+^ T-cell responses along with the induction of NK cell-mediated immunity in mice [77]. 

Overall, the final outcome of HSP^+^ exosomes on NK cell anti-tumor immune response depends on a direct stimulatory effect on these cytotoxic lymphocytes and/or on the different subset of immune cells localized in the tumor microenvironment and responsive to HSPs (Figure 1b).

### 4.3. MicroRNAs

MicroRNAs (miRNAs) are a class of small (19–25 nucleotides) non-coding single-stranded RNA molecules that have emerged as key players in the post-transcriptional regulation of protein expression and degradation. These small RNAs have been described to be a crucial component of exosomal cargo [79] and specific sets of miRNAs are enriched into exosomes thanks to specific molecular sorting mechanisms due to the involvement of RNA binding proteins [80,81,82,83]. In this regard, Villarroya-Beltri and colleagues observed that the sumoylation of the heterogeneous nuclear ribonucleoprotein A2B1 (hnRNPA2B1) regulates the binding to a miRNA motif, thereby affecting miRNA exosomal loading [81]. In both mice and humans, miRNAs have been shown to be critical regulators of NK cell activation, survival and function [84,85]. Cellular stress can strongly affect the exosomal miRNA cargo. For example, Umezu and colleagues have shown that the amount of miR-135b associated with multiple myeloma-derived exosomes increased in response to hypoxia [86]. Similarly, breast cancer cells cultured in hypoxic conditions released EVs carrying miR210 that inhibited NK cell-mediated functions [87].

In addition to the classical role of miRNAs able to specifically target mRNAs determining their degradation or the inhibition of their translation, mounting evidence has shown that selected miRNAs can act as DAMPs by engaging TLRs, thus suggesting an alternative mechanism of innate immune cell regulation [88,89,90,91]. As such, Fabbri and colleagues demonstrated that miR-21 and miR-29a, highly expressed into lung cancer derived exosomes, can directly bind to TLR8 in human macrophages thereby causing proinflammatory cytokine production (i.e., IL-6, TNF-α) through the activation of the NF-κB pathway [88]. Recently, another study has reported that some exosomal miRNAs possessing an IFN induction motif (IIM), such as miR-574, let-7b and miR-21, can act as endogenous ligands of TLR7, leading to the activation of plasmacytoid DCs [91]. 

Altogether, these findings show that miRNAs profile into exosomes can change in response to stress conditions and in some circumstances, specific miRNAs, through the direct binding to TLRs, act as danger signals, stimulating innate immune cells (Figure 1c).

### 4.4. Cytosolic DNA

In normal conditions DNA is confined to the nucleus and mitochondria; however, in the presence of DNA damage, DNA accumulates in the cytoplasm where it is detected as a DAMP by a number of cytosolic sensors that converge on the STING (stimulator of IFN gene) adaptor protein pathway leading to type I interferon (IFN) expression. Cyclic guanosine monophosphate (GMP)-adenosine monophosphate synthase (cGAS) represents a key cytosolic sensor able to detect dsDNA into the cytoplasm [92]. Upon cGAS DNA binding, this enzyme produces a second messenger, the cyclic 2′3′-GMP-AMP (cGAMP) that represents a high affinity ligand for STING [92,93,94]. Actually, it is largely unclear how DNA damage leads to DNA accumulation in the cytosol. A number of recent studies report that nuclear DNA in the cytoplasm can be loaded and stored into exosomes [21,22,95,96,97]. In general, DNA associated with cancer-derived exosomes is more abundant when compared to healthy cells [22]. Takahashi and colleagues have demonstrated that the inhibition of exosome secretion results in the accumulation of nuclear DNA in the cytoplasm, thereby causing the activation of cGAS-STING pathway leading to type I IFN production [95]. These data indicate that exosome secretion might maintain cellular homeostasis by removing harmful cytoplasmic DNA from cells. Another recent study has shown that tumor-derived exosomes produced by irradiated mouse breast cancer cells transfer dsDNA to DCs and stimulate upregulation of cell surface costimulatory molecules, as well as the STING-dependent activation of type I IFNs [98]. Similarly, the anti-tumor agent topotecan (TPT), an inhibitor of topoisomerase I, has been shown to stimulate the release of exosomes containing DNA from breast cancer cells, thus leading to DC activation via STING signaling [96]. Overall, these findings show that dsDNA associated with cancer-derived exosomes triggers type I IFN production directly from cancer cells or indirectly through the DCs stimulation (Figure 1d). Accumulating evidence suggests that type I IFNs have a crucial role in cancer progression through the promotion of anti-cancer immune responses [99,100]. In particular, it has been observed that type I IFNs enhanced DC-mediated cross-presentation of tumor antigens to cytotoxic T lymphocytes [99,101,102]. In addition, type I IFNs promote DCs to release IL-15, which is important to maintain the survival of CD8+ memory cells and NK cells [103,104]. Type I IFNs can directly activate NK cell-mediated functions increasing perforin-dependent cytotoxicity [105] and inducing TRAIL expression; moreover, with the coordinated action of IL-12, type I IFNs greatly enhance NK cell-mediated IFN-γ production. The possible effects of dsDNA associated with Tex to modulate NK cell activity is depicted in Figure 1d.

## 5. Conclusions and Perspectives

The capability of cancer-derived exosomes to carry immunomodulatory molecules represents a crucial aspect in the anti-tumor immune response, although further studies are necessary to evaluate the in vivo role of cancer-derived exosomes. Mounting evidence reveals that exosomes secreted from cancer cells in response to chemotherapeutic treatments and, in general, to stress conditions, represent important messengers of DAMPs, as well as stress-induced molecules such as NKG2D ligands. It is still largely unknown whether exosomal-associated NKG2D ligands function differently from those released through protease-mediated cleavage. Indeed, exosomes transport simultaneously distinct biologically active molecules that can cooperate to trigger together signals to the target cell. Furthermore, a better characterization of exosome molecular phenotype and immunomodulatory properties will provide new insights into their immune-regulatory role during the course of chemotherapeutic interventions and, possibly, into their use as prognostic biomarkers.
